# Genetic diversity of the rain tree (*Albizia saman*) in Colombian seasonally dry tropical forest for informing conservation and restoration interventions

**DOI:** 10.1002/ece3.6005

**Published:** 2020-02-05

**Authors:** Carolina Adriana Aguirre‐Morales, Evert Thomas, Carlos Ivan Cardozo, Janeth Gutiérrez, Carolina Alcázar Caicedo, Luis Gonzalo Moscoso Higuita, Luis Augusto Becerra López‐Lavalle, Mailyn Adriana González

**Affiliations:** ^1^ Universidad Nacional de Colombia Sede Palmira Palmira Colombia; ^2^ Bioversity International Lima Peru; ^3^ International Center for Tropical Agriculture Cali Colombia; ^4^ Bioversity International Cali Colombia; ^5^ Forestpa SAS Medellín Colombia; ^6^ Instituto de Investigación de Recursos Biológicos Alexander von Humboldt Bogotá Colombia

**Keywords:** agroforestry, climate change, microsatellites, paleodistribution, seed zones, suitability modeling

## Abstract

*Albizia saman* is a multipurpose tree species of seasonally dry tropical forests (SDTFs) of Mesoamerica and northern South America typically cultivated in silvopastoral and other agroforestry systems around the world, a trend that is bound to increase in light of multimillion hectare commitments for forest and landscape restoration. The effective conservation and sustainable use of *A. saman* requires detailed knowledge of its genetic diversity across its native distribution range of which surprisingly little is known to date. We assessed the genetic diversity and structure of *A.saman* across twelve representative locations of SDTF in Colombia, and how they may have been shaped by past climatic changes and human influence. We found four different genetic groups which may be the result of differentiation due to isolation of populations in preglacial times. The current distribution and mixture of genetic groups across STDF fragments we observed might be the result of range expansion of SDTFs during the last glacial period followed by range contraction during the Holocene and human‐influenced movement of germplasm associated with cattle ranching. Despite the fragmented state of the presumed natural *A. saman* stands we sampled, we did not find any signs of inbreeding, suggesting that gene flow is not jeopardized in humanized landscapes. However, further research is needed to assess potential deleterious effects of fragmentation on progeny. Climate change is not expected to seriously threaten the in situ persistence of *A. saman* populations and might present opportunities for future range expansion. However, the sourcing of germplasm for tree planting activities needs to be aligned with the genetic affinity of reference populations across the distribution of Colombian SDTFs. We identify priority source populations for i*n situ* conservation based on their high genetic diversity, lack or limited signs of admixture, and/or genetic uniqueness.

## Graphical abstract

Through the combination of spatial analysis of genetic characterization data with the outcomes of suitability modeling under past climates, we aim to understand how past climate change and human influences can help explaining contemporary patterns in genetic diversity of Albizia saman, a tree species typically used in agroforestry systems around the world. Our findings suggest that the genetic structure of A. saman populations may partly be explained by the dry forest refugia hypothesis and that  more recent human‐influenced movement of germplasm associated with cattle ranching resulted in profound admixing of most populations. We make a number of recommendations to guide the sourcing of germplasm for future planting endeavors and identify priority areas for in situ conservation of populations.

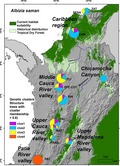

## 1 INTRODUCTION

**Figure 1:**
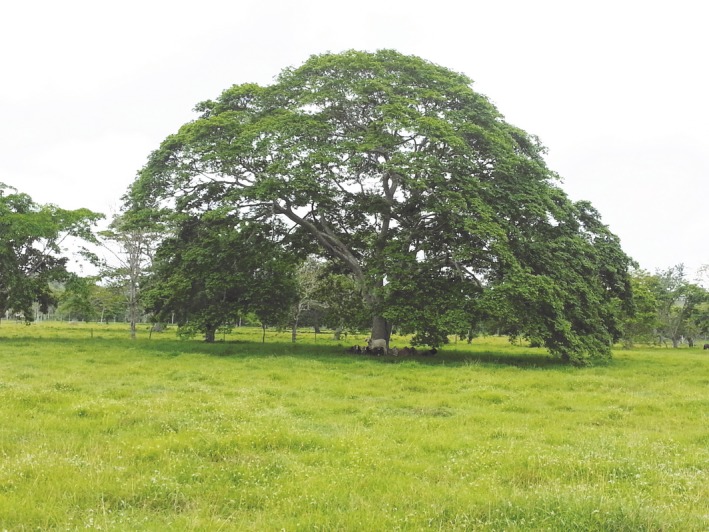
Typical use of *Albizia saman* as shade and fodder tree in pasture land in Colombia

**Figure 2:**
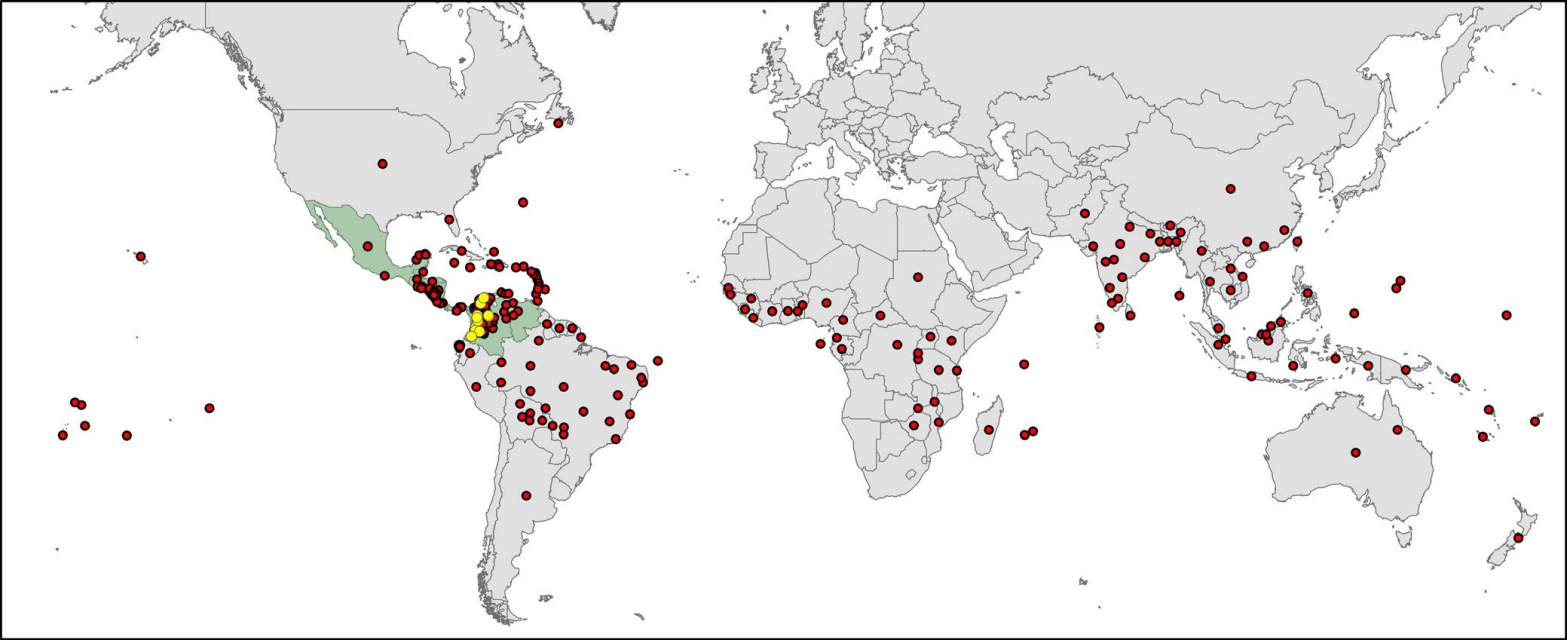
Global distribution of *Albizia saman* (red dots). Its native area is believed to be restricted to the region from southern Mexico to Colombia and Venezuela, but it has been introduced to tropical areas all around the world**.** The countries that are believed to be part of the native range of the species are shown in green, and the locations of the trees sampled in the current study are shown as yellow dots

*Albizia saman* (Jacq.) Merr*.* (Fabaceae) is a multipurpose tree species occurring naturally in the seasonally dry tropical forests (SDTF) from southern Mexico to Colombia and Venezuela (Cascante, Quesada, Lobo, & Fuchs, 2002; Durr, 2001). It is valued for its edible fruit pulp and medicinal properties (Leonard & Sherratt, 1967) and the production of an exudate with industrial applications (Subansenee, 1994), whereas its wood is exploited for manufacturing furniture and crafts (Escalante, 1997). However, the by far most important use of this wide‐canopied tree is in agroforestry systems (Figure 1), owing to its rapid growth, the shade provided by its thick foliage, the nutrient‐rich fodder produced by its leafs and fruits, and positive effects on the productivity of soils and grazing land (Allen & Allen, 1981; Durr, 2001; Roshetko, 1995). These useful traits have been a major motivation for the introduction of *A. saman* from its native distribution in Central and northern South America to other tropical areas in the Americas and the rest of the world, where it has often become naturalized (CABI, 2018) (Figure 2).

The sustainable use and effective conservation of *A. saman* requires detailed knowledge of the species' genetic diversity across its native distribution range of which surprisingly little is known to date. Only one study investigated the effects of fragmentation on the reproductive success and genetic structure of the species in northwestern Costa Rica (Cascante et al., 2002). While a better understanding of population responses to threats like fragmentation is essential for guiding conservation and management interventions, it needs to be supplemented with information on genetic differentiation of populations across their distribution ranges.

Aside from the idiosyncrasies of their life history traits and reproductive biology (Duminil et al., 2007; Lowe et al., 2018), the contemporary genetic structure of tree species is influenced most notably by their response to past changes in climate and more recent anthropogenic disturbances. One of the most important impacts of climate change in the recent past on neotropical SDTFs is conceptualized through the dry forest refugia hypothesis (DFRH) (Mayle, Beerling, Gosling, & Bush, 2004; Pennington, Prado, & Pendry, 2000; Prado & Gibbs, 1993). The DFRH postulates that the current wide distribution of numerous tree species in disjointed areas of SDTF is the result of the contraction of an extensive and continuous formation of the biome during the last glacial period (18,000–12,000 BP) to the remnants observed today (Pennington et al., 2000; Prado & Gibbs, 1993).

The postglacial isolation of tree species populations in different SDTF fragments is likely to have initiated processes of genetic differentiation but seems to have been too short to be detected in the current population genetic structures. An increasing body of evidence suggests that the formation of different genetic groups in SDTF tree species may predate the late Pleistocene (Bocanegra‐González et al., 2018; Caetano et al., 2008; Collevatti et al., 2012; Vitorino, Lima‐Ribeiro, Terribile, & Collevatti, 2016). According to the DFRH, one would thus expect a similar disjointed distribution of genetic groups within tree species present in different SDTF fragments that used to be connected during the last glacial period. Here, we test this hypothesis for *A. saman* in Colombian SDTF.

Tree species populations in SDTF fragments have been subject to anthropogenic disturbance since pre‐Columbian times (Banda‐R et al., 2016; Murphy & Lugo, 1986). Conversion of mature SDTF in Colombia to land for human settlements and crop production and pastures for cattle ranching intensified during the European colonization period and particularly so during the past century (Etter, 2015; Vina & Cavelier, 1999). Today, STDF is one of the most threatened ecosystems worldwide (Janzen,^1988^; Miles et al., 2006). In Colombia, less than 8% of the original STDF cover remains, occurring in a highly fragmented state (García, 2014; González‐M et al., 2018). Particularly in predominantly outcrossing species such as *A. saman*, fragmentation of populations is known to negatively affect the reproduction, gene flow, and genetic diversity of tree populations, resulting in increased risk of inbreeding depression in progeny and loss of genetic diversity and fitness due to low numbers of mating partners and low pollen diversity (Aguilar, Ashworth, Galetto, & Aizen, 2006; Aguilar, Quesada, Ashworth, Herrerias‐diego, & Lobo, 2008; Lowe, Boshier, Ward, Bacles, & Navarro, 2005).

Here, we elucidate the genetic diversity, structure, and inbreeding state of *A saman* populations across the main SDTF fragments in Colombia, located in the Caribbean region and the Cauca, Magdalena, Chicamocha, and Patia river valleys (Pizano, Cabrera, & García, 2014), and relate these to the effects of past climate change and anthropogenic disturbance. Based on our findings, we identify priority areas for in situ conservation of *A. saman* and make some recommendations on the potential use of populations as seed sources in future tree planting efforts.

## 2 METHODS

### 2.1 Field sampling

We collected leaf material from 100 reproductive individuals of *A. saman* between July 2014 and January 2016 across twelve representative locations of STDF in Colombia. Sampled trees were separated by at least 50 m to avoid the collection of highly genetically related individuals (Gonzalez & Quintero, 2017). All biological materials were collected in collaboration with the Instituto Alexander von Humboldt following the Colombian Decreto 302 of 2003.

### 2.2 DNA extraction and PCR amplification

Young and healthy leaves of sampled *A. saman* trees were preserved in paper bags and dried with silica gel prior to processing in the laboratory. Total genomic DNA was isolated from dried plant material using 80 mg of leaf tissue in accordance with the CTAB method (Doyle & Doyle, 1990) with modifications following Alzate‐Marin, Guidugli, Soriani, Martinez, & Mestriner, 2009; Novaes, Rodrigues, & Lovato, 2009; Verbylaite, Beisys, Rimas, & Kuusiene, 2010. Genetic characterization was carried out by means of twelve specific microsatellite markers (Kasthurirengan, Xie, Li, Fong, & Hong, 2013). Each PCR reaction was carried out in a total volume of 15 μl containing 1× PCR buffer of 200 mM Tris‐HCl (pH 8.4), 500 mM KCl (Invitrogen^®^, USA), 0.25 mM dNTP (Promega Corp., USA), 4 mM MgCl_2_ (Invitrogen^®^, USA), pmol/μl M13 tagged forward primer 0.1 and 0.2 pmol/μl reverse primer, 0.15 pmol/μl universal fluorescent‐labeled M13 primer, 0.03 U Platinum^®^ Taq (Invitrogen^®^, USA) and 40 ng of DNA, following Schuelke (2000). The master mix was complemented with bovine serum albumin (BSA 3%) and/or formamide 2.5%. PCR amplifications were performed in Eppendorf Mastercycler*^®^* pro (Eppendorf, Germany) with an initial cycle of 2 min at 95°C followed by 15 cycles of 30 s at 94°C, 30 s at 65°C, and 30 s at 72°C, and finally 35 cycles of 15 s at 94°C, 15 s at 50°C, and 45 s at 72°C. PCR products were run on an ABI PRISM 3730 DNA Analyzer sequencer and sized with GeneScan 500LIZ (Applied Biosystems) standard size. Allele sizes were determined using GeneMapper version 4.0 (Applied Biosystems).

### 2.3 Diversity mapping and genetic structure

We visualized geographic patterns in nSSR diversity on raster maps of 30 arc seconds resolution by constructing circular neighborhoods of 10 arc minutes diameter (~18 km at the equator) around the locations of all the genotyped *A. saman,* following Thomas et al. (2012). In practice, this means that each tree was replicated in all the 30 arc second grid cells contained in a circle with diameter of 10 arc minutes constructed around its location. As this replication exercise resulted in different numbers of trees per grid cell, in a next step we performed a sample bias correction by calculating genetic parameters as the average values obtained from 1,000 bootstrapped subsamples of the minimum sample size of 3 trees per grid cell. Grid‐based calculations of genetic parameters included allelic richness, the Shannon information index, expected and observed heterozygosity, the inbreeding coefficient, and the number of locally common alleles (LCA) per locus. LCA are alleles that are restricted to a limited area of a species' distribution (here < 25% of the sampled populations) but reach relatively high frequencies (here > 5%) in those areas. High LCA richness can be indicative for the level of genetic isolation of populations (Frankel, Brown, & Burdon, 1995a) and can hence be helpful for identifying putative refugia (Marchelli, Thomas, Azpilicueta, Zonneveld, & Gallo, 2017; Thomas et al., 2012).

We submitted our data to Bayesian cluster analysis in STRUCTURE (Pritchard, Stephens, & Donnelly, 2000) using an admixture ancestry model without consideration of sampling localities. The number of groups (*K*) tested varied between 1 and 8, using burnin periods of one million steps and 10 million additional replications. For each value of *K*, we carried out 10 independent repetitions. We used the method of Evanno, Regnaut, and Goudet (2005) for detection of the most probable number of genetically homogeneous clusters (*K*), through calculation of Δ*K* as implemented in the STRUCTURE HARVESTER software (Dent & VonHoldt, 2011). Complementary genetic analyses such as F_ST_ (Nei, 1973) and AMOVA (Excoffier, Smouse, & Quattro, 1992) were carried out in R packages *adegenet* (Jombart, 2008) and *poppr* (Kamvar, Tabima, & Grünwald, 2014).

### 2.4 Suitability modeling

**Figure 3:**
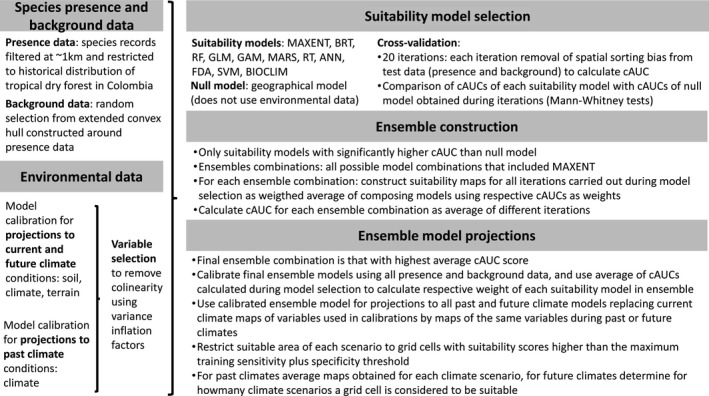
Summary of the modeling procedure used for identifying suitable habitat for *Albizia saman* in Colombian seasonally dry tropical forests during different past present and future climate scenarios

We characterized the spatial distribution of favorable habitat for *A. saman* in Colombian SDTFs under different climatic conditions by means of suitability mapping based on ensembles of modeling algorithms, implemented in R package BiodiversityR (Kindt, 2018). Figure 3 summarizes the modeling procedure used. We modeled habitat suitability during the LGM (~21,000 BP) and mid‐Holocene (~6,000 BP) to assess potential impacts of past climate conditions on the current distribution of genetic diversity. We similarly modeled habitat suitability under present and future climate conditions to evaluate the expected impact of climate change on the in situ persistence of *A. saman* populations.

Presence data collected during our field sampling were complemented with Colombian records extracted from numerous sources (http://www.gbif.org; the national herbaria MEDEM, HUA, MEDEL, COL, CUVC, VALLE and TULV; http://www.dryflor.info; http://www.orinoquiabiodiversa.org; http://www.sibcolombia.net). We only included records located in SDTF as defined by the combination of Etter, McAlpine, and Possingham (2008) and García et al. (2014). As a result, 151 unique presence points were used for suitability modeling. Background points (an overall maximum of 10,000 and maximum one per grid cell) were randomly selected from the area enclosed by a convex hull polygon constructed around all presence points and extended with a buffer corresponding to 10% of the polygon's largest axis. We applied two different strategies for suitability modeling under past and future climate conditions. Model calibrations for projections to LGM and mid‐Holocene climate conditions were carried out at 2.5 arc minutes and 30 arc seconds resolution, respectively, using only WorldClim climate layers (Hijmans, Cameron, Parra, Jones, & Jarvis, 2005) as explanatory variables. Model calibrations intended for projections to future climate scenarios (period 2040–2069; referred to as 2050s) were carried out at 30 arc seconds resolution, using aside from climate layers also altitude, slope, aspect, terrain roughness, direction of water flow, and seven major edaphic variables, obtained from ISRIC‐World Soil Information (Hengl et al., 2014): organic carbon (ORCDRC), pH in H_2_O (PHIHOX), sand % (SNDPPT), silt % (SLTPPT), clay % (CLYPPT), cation exchange capacity (CEC), bulk density (BLD), and coarse fragments > 2 mm (CRFVOL). For the edaphic variables, we calculated a weighted mean across 0–5, 5–15, 15–30, 30–60, and 60–100 cm soil depth values in order to derive a single data value for 0–100 cm. Weights were proportional to the thickness of each soil layer (i.e., 0.05, 0.1, 0.15, 0.3, and 0.4). Collinear explanatory variables were removed based on iterative calculations of variance inflation factors (VIF), retaining only variables with VIFs smaller than 5. VIFs were calculated using self‐assembled R script. The resulting sets of explanatory variables, as well as presence and background points used for model calibrations, are given in Table S5 of the Electronic Supplementary Material.

Modeling algorithms considered in the ensembles were maximum entropy (MAXENT), boosted regression trees (BRT; including a stepwise implementation), random forests (RF), generalized linear models (GLM; including stepwise selection of explanatory variables), generalized additive models (GAM; including stepwise selection of explanatory variables), multivariate adaptive regression splines (MARS), regression trees (RT), artificial neural networks (ANN), flexible discriminant analysis (FDA), support vector machines (SVM), and the BIOCLIM algorithm. The ensemble modeling was carried out using the BiodiversityR package using default settings. The specifics of how different models are implemented are explained in the package help function and Kindt (2018). As spatial autocorrelation among species presence points is known to bias model evaluations based on cross‐validation, we evaluated the ability of all individual modeling algorithms to cope with spatial autocorrelation by calculating calibrated Area Under Curve (cAUC) values and comparing these with a geographical null model (Hijmans, 2012). We compared the cAUCs of each of the individual distribution models with the cAUCs of the geographical null model resulting from twenty iterations, by means of Mann–Whitney tests. Only models that gave cAUC values that were significantly higher than the null model were retained for the construction of different model ensembles (Tables S1 and S2). In a next step, we calculated the cAUC values for all possible ensemble combinations of the retained models that included MAXENT, which is generally considered a superior suitability model (Tables S3 and S4). This resulted in 1,024 possible ensemble combinations for projections to current and future climate conditions and 8,192 possible ensemble combinations for projections to past climate conditions. Each ensemble combination was constructed as the weighted average of its individual composing models, using their respective average cAUC values as weights. The ensemble that yielded the highest cAUC value was considered to generate the most appropriate scenario for projecting to past and future climate conditions, respectively (Table S5).

To assess habitat suitability under mid‐Holocene and LGM climate conditions, we carried out projections to two and three climate models, respectively (BCC‐CSM1‐1 and CCSM4, and MIROC‐ESM, MPI‐ESM‐P, and CCSM4). For characterizing future climate conditions, we used 31 downscaled climate models for the period 2040–2069 based on the representative concentration pathway (RCP) 4.5 scenario of greenhouse gas emissions, prepared for the Fifth Assessment IPCC report (CMIP5) (Ramirez Villegas & Jarvis, 2010). We limited model projections to areas where suitability scores were higher than the maximum training sensitivity plus specificity threshold obtained from model calibration under current climate conditions. To obtain summarizing maps for the LGM and mid‐Holocene climate models, we averaged the threshold‐limited suitability maps constructed for both individual climate scenarios. Two scenarios were considered for future suitability maps. Optimistic and pessimistic scenario maps were limited to areas which were identified as suitable by at least one, and half of all 31 possible threshold‐limited climate projections, respectively. All maps were edited in ArcMap 10.2

## 3 RESULTS

**Figure 4:**
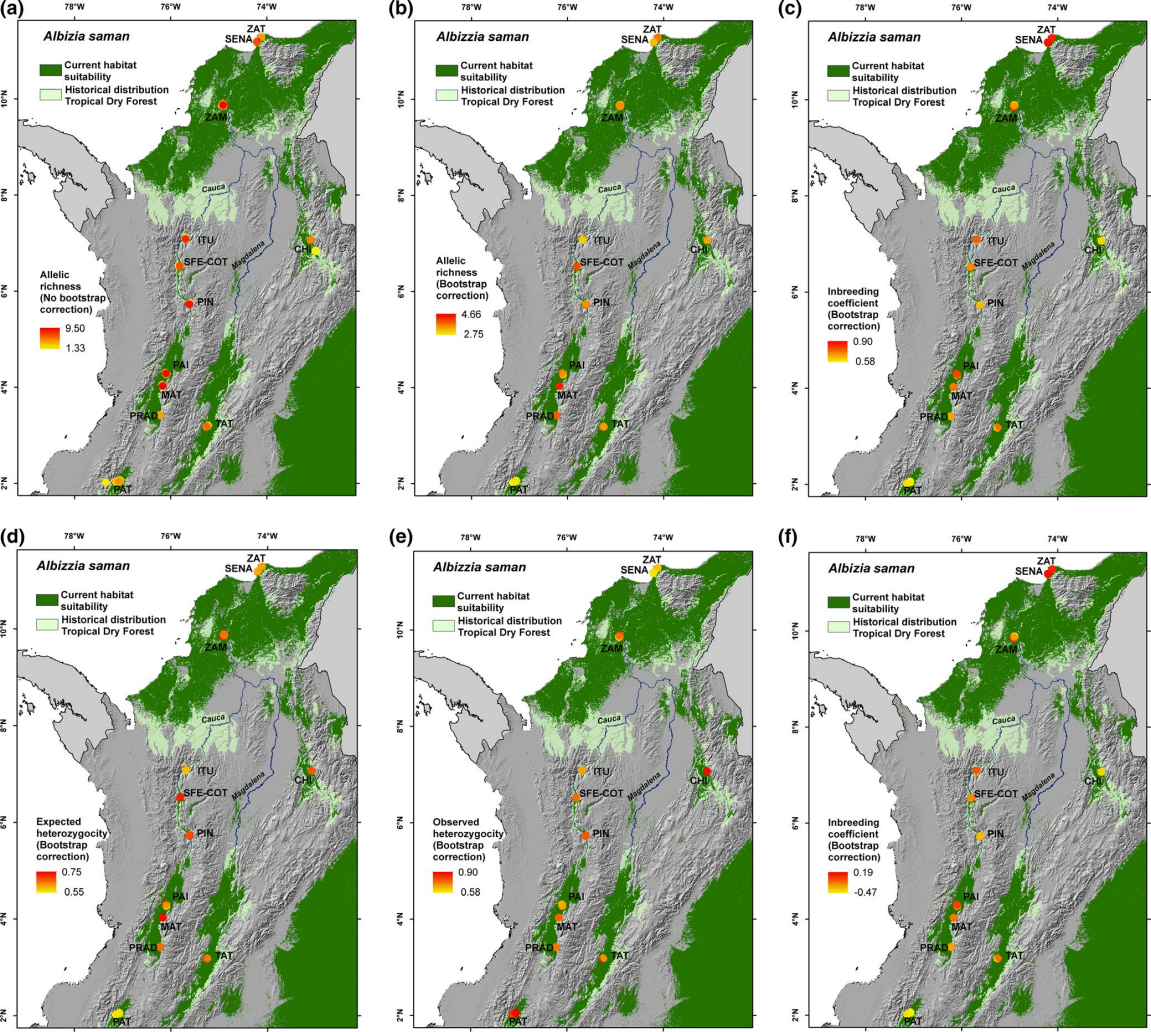
Spatial variation of different genetic parameters of *Albizia saman* against a background of the species' modeled habitat suitability under current climate conditions (dark green), and the historical distribution of seasonally dry tropical forest in Colombia (light green): (a) allelic richness without sample bias correction; (b) allelic richness with bootstrap correction; (c) Shannon index with bootstrap correction; (d) expected heterozygosity with bootstrap correction; (e) observed heterozygosity with bootstrap correction; and (f) inbreeding coefficient with bootstrap correction

All twelve microsatellite markers yielded highly variable allele numbers per locus, ranging from 13 to 28 alleles. Overall, different genetic diversity measures (allelic richness, Shannon diversity, and expected heterozygosity) suggest that most of the sampled populations hold comparable levels of diversity (Table 1). The lowest values were observed in the isolated Patia (PAT) population and the highest in the upper (MAT) and middle Cauca river valley (SFE‐COT). The inbreeding coefficient varied from −0.26 to 0.23, but only values found for SENA (*F*_IS_ = 0.23) and Patia (PAT; *F*_IS_ = −0.26) differed significantly from Hardy‐Weinberg expectations (*p* < .05; Table 1). Figure 4 shows the spatial distribution of the genetic diversity parameters against a background of the species´ current habitat suitability and the historical distribution of SDTF in Colombia.

**Table 1:** Genetic parameters of *Albizia saman* estimated in 12 sampling sites located across Colombian seasonally dry tropical forests

Regions	ID	*n*	Uncorrected values					Bootstrap corrected values				
			A	I	He	Ho	*F*_IS_	A	I	He	Ho	*F*_IS_
Caribbean region	ZAT	**3**	**4.08**	**1.30**	**0.69**	**0.67**	**0.09**	**4.08**	**1.30**	**0.69**	**0.67**	**0.09**
			1.31	0.37	0.13	0.35	0.42	1.31	0.37	0.13	0.35	0.42
	SENA	**7**	**7.08**	**1.79**	**0.81**	**0.62**	0.23**	**3.94**	**1.27**	**0.69**	**0.62**	**0.10**
			1.93	0.30	0.06	0.19	0.25	0.97	0.28	0.10	0.29	0.40
	ZAM	**14**	**9.50**	**1.99**	**0.83**	**0.75**	**0.07**	**4.05**	**1.30**	**0.70**	**0.75**	**−0.08**
			2.84	0.40	0.08	0.20	0.29	0.90	0.26	0.09	0.28	0.40
Cauca River valley	ITU	**10**	**8.08**	**1.88**	**0.82**	**0.70**	**0.13**	**3.89**	**1.26**	**0.68**	**0.70**	**−0.03**
			2.07	0.27	0.05	0.25	0.32	1.02	0.29	0.10	0.33	0.47
	SFE‐COT	**7**	**7.17**	**1.82**	**0.81**	**0.76**	**0.06**	**4.24**	**1.36**	**0.72**	**0.76**	**−0.06**
			1.40	0.22	0.04	0.21	0.29	0.87	0.23	0.07	0.27	0.37
	PIN	**12**	**8.75**	**1.95**	**0.83**	**0.77**	**0.06**	**4.05**	**1.31**	**0.70**	**0.77**	−0.10**
			2.73	0.30	0.05	0.19	0.25	0.95	0.27	0.09	0.27	0.38
	PAI	**12**	**9.17**	**1.96**	**0.82**	**0.70**	**0.14**	**4.05**	**1.30**	**0.69**	**0.70**	**−0.01**
			2.72	0.32	0.06	0.24	0.30	1.00	0.29	0.10	0.31	0.43
	MAT	**10**	**9.08**	**2.03**	**0.85**	**0.77**	**0.09**	**4.38**	**1.39**	**0.73**	**0.77**	**−0.05**
			1.73	0.22	0.04	0.22	0.26	0.91	0.25	0.08	0.29	0.38
	PRAD	**3**	**4.17**	**1.32**	**0.70**	**0.75**	**−0.09**	**4.17**	**1.32**	**0.70**	**0.75**	**−0.09**
			1.11	0.31	0.10	0.29	0.42	1.11	0.31	0.10	0.29	0.42
Chicamocha	CHI	**5**	**5.50**	**1.58**	**0.77**	**0.84**	**−0.11**	**4.03**	**1.31**	**0.70**	**0.84**	**−0.20**
cañon			1.88	0.34	0.08	0.25	0.35	1.04	0.28	0.08	0.27	0.40
Magdalena	TAT	**6**	**6.42**	**1.69**	**0.78**	**0.72**	**0.06**	**4.04**	**1.30**	**0.70**	**0.72**	**−0.05**
River valley			1.62	0.30	0.07	0.22	0.35	0.91	0.26	0.09	0.28	0.42
Patia	PAT	**11**	**4.67**	**1.31**	**0.69**	**0.85**	−0.26*	**3.18**	**1.04**	**0.61**	**0.85**	−0.41**
River valley			1.07	0.24	0.08	0.19	0.35	0.85	0.27	0.10	0.25	0.41

Note

**Figure 5:**
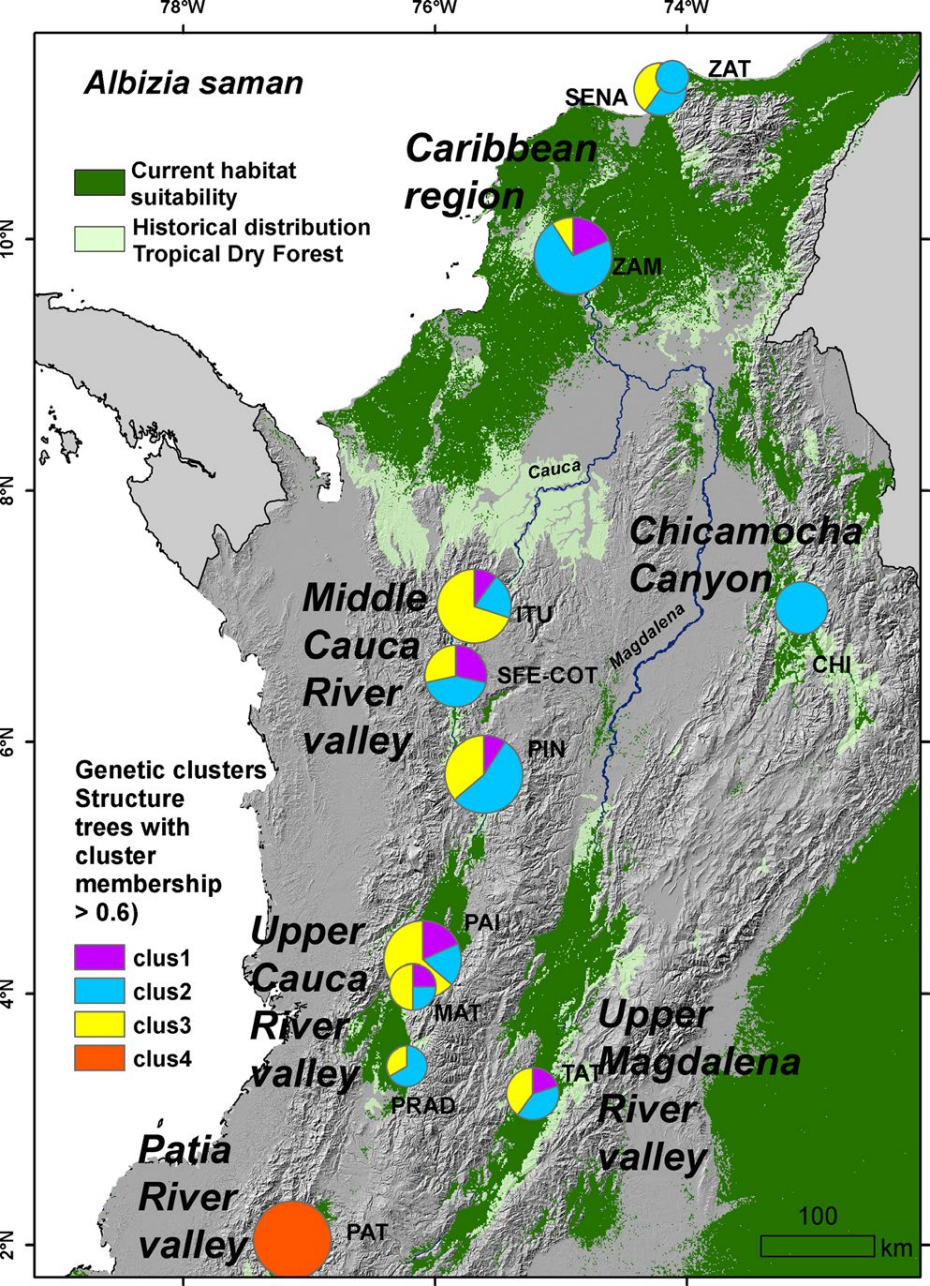
Distribution of genetic groups across sampled populations of *Albizia saman* against a background of the species' modeled habitat suitability under current climate conditions (dark green), and the historical distribution of seasonally dry tropical forest in Colombia (light green). Circles sizes are proportionate to the number of individuals

For locations of the regions and sampling sites (IDs), please refer to Figure 5. All values are multilocus estimates based on 12 microsatellite loci. Values in bold are parameter estimates and values in regular font type are standard deviations.

ID abbreviation of sampling sites; *n* sample sizes; A allelic richness; I Shannon diversity; He expected heterozygosity; Ho observed heterozygosity; and *F*_IS_ multilocus estimate of the inbreeding coefficient.

* and ** significantly different from 0 at *p* < .05 and .01, respectively.

Analysis of molecular variance (AMOVA) indicated that 10.6% of the total genetic variation resided between populations, compared with 89.4% between individuals within populations. Pairwise F_ST_ values between sampling areas were generally low, with the exception of Patia which yielded a mean F_ST_ value of 0.14, which was twice as high as the population with the second highest mean value (ZAT, *F*_ST_ = 0.07; Table S6).

Analyses carried out in *STRUCTURE* showed support for two highly differentiated genetic clusters (*K* = 2): One grouping trees sampled in the Patía river valley (PAT) and another one grouping all individuals from the rest of the country (Figure S1a). However, Δ*K* computation also showed support for *K* = 4, identifying 3 different subclusters in the second group (Figure S1a). Repeating the analysis with the exclusion of Patía samples similarly resulted in support for *K* = 3 (Figure S1b). Most of the sampling sites outside of Patia were composed of individuals assigned to two or three different clusters (Figure S1c; Figure 5). It is important to note here that due to the modest sampling sizes at some sampling sites, signals of potential genetic differentiation have to be interpreted with caution.

**Figure 6:**
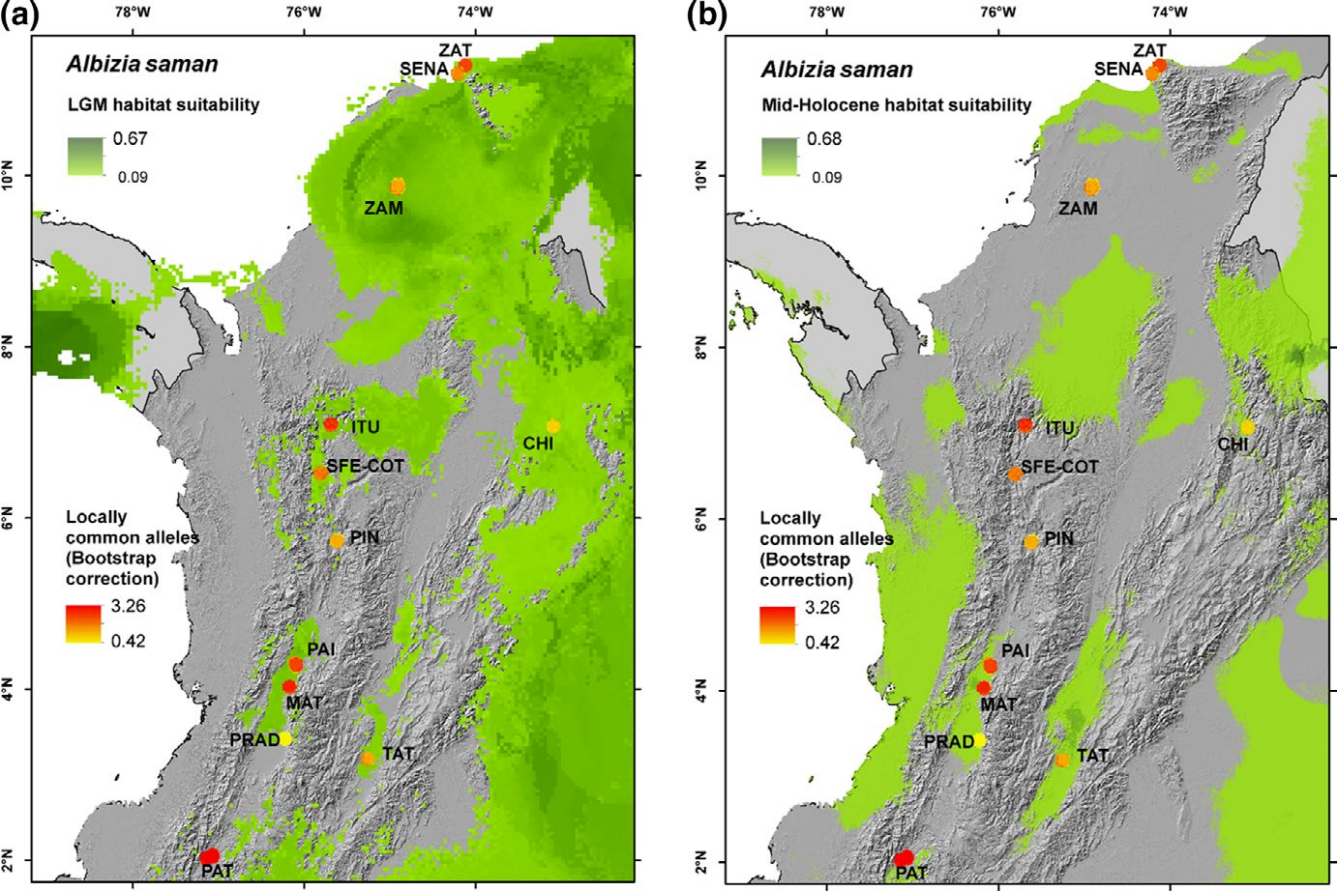
Modeled distribution of suitable habitat of *A. saman* during (a) the Last Glacial Maximum (LGM; ~21,000 BP) and (b) the mid‐Holocene (~6,000 BP), in combination with richness of locally common alleles (LCA)

The modeled distributions of suitable habitat during past climates (Figure 6) suggest that *A. saman* populations from SDTF in Colombia may have had a much wider range during the LGM than at present and that the current disjunct distribution patterns may have started to form in the early Holocene. The LGM map suggests that suitable habitat conditions were found in a large continuous area connecting the Caribbean region with the Chicamocha Canyon, and possibly the upper Magdalena river valley. Figure 6a,b also suggest that *A. saman* populations from the Patia, upper and middle Cauca river valleys have been isolated from each other from the LGM or longer until at least the mid‐Holocene. The highest values of LCA richness were found in locations that overlap with, or are adjacent to, areas sustaining suitable habitat conditions for *A. saman* during different periods of time since the LGM, that is ZAT, ITU, PAI & MAT, and PAT (Figure 5a,b).

**Figure 7:**
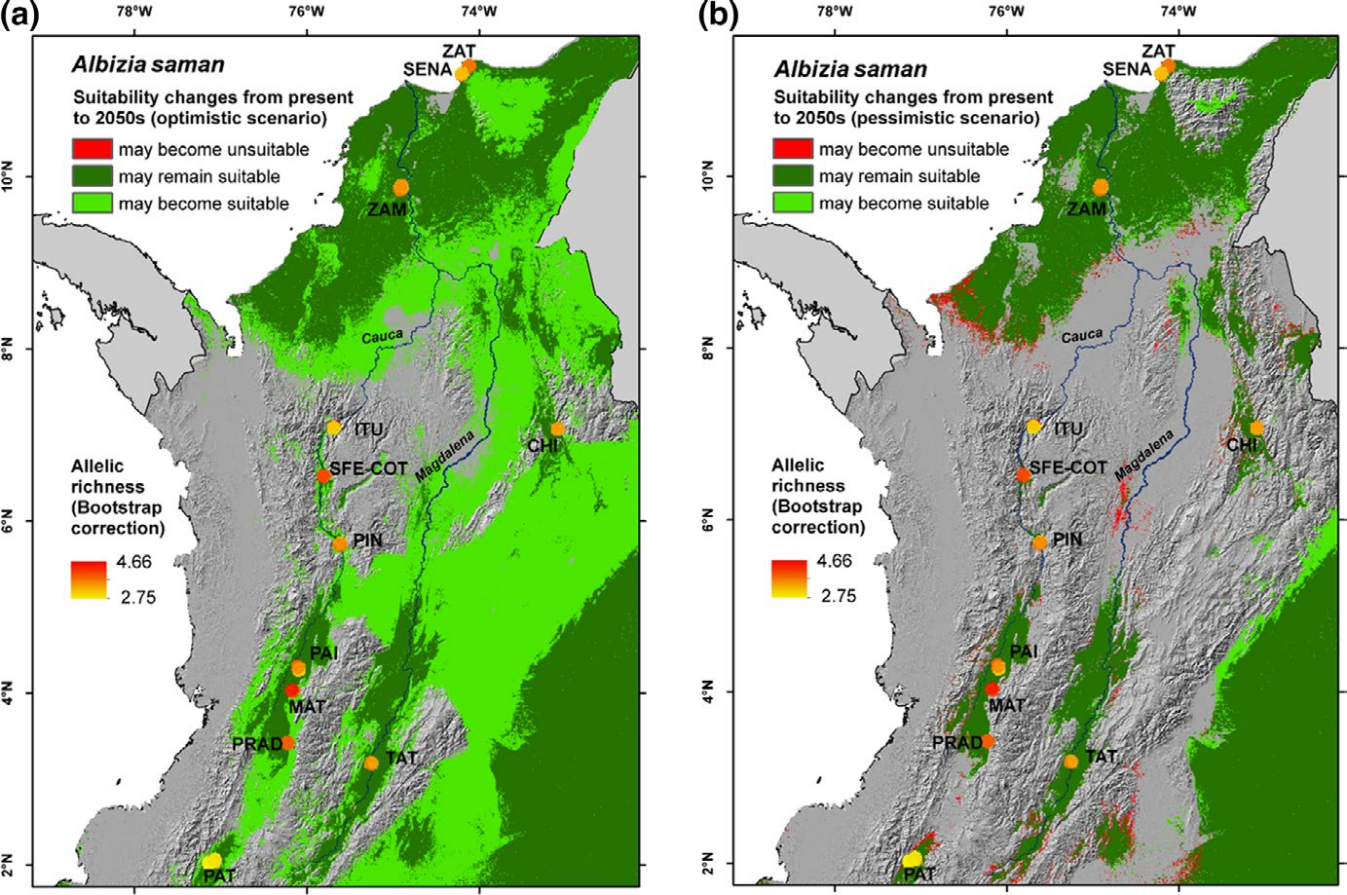
Future (2050s) habitat suitability of *A. saman* showing all areas identified as suitable by at least 1 (a) and 15 (b) of 31 future climate model projections in combination with the distribution of allelic richness

Future climate projections for the period 2040–2069 (Figure 7) suggest that nearly all currently suitable areas of *A. saman* are likely to remain so according to at least half of the 31 climate models we considered (Figure 7b) and might significantly expand in the near future according to smaller subsets of models (Figure 7a).

## 4 DISCUSSION

We assessed the genetic diversity distribution of *A. saman* populations across Colombian SDTF fragments and how it may have been shaped by past climatic changes and more recent human influences. Our habitat suitability models during the LGM and mid‐Holocene are consistent with the DFRH (Mayle et al., 2004; Pennington et al., 2000; Prado & Gibbs, 1993). Joint interpretation of modeling results and the genetic characterization data gives clues about the origin of the four genetic groups we identified in *A. saman* and suggests that the genetic differentiation of these groups is likely to predate the LGM, in line with similar findings for other SDTF species (Bocanegra‐González et al., 2019, 2018; Caetano et al., 2008; Collevatti et al., 2012; Thomas et al., 2017b; Vitorino et al., 2016).

The four genetic clusters of *A. saman* in Colombian SDTF are paralleled by four locations which showed peaking LCA richness values (possibly indicative of prolonged genetic isolation of populations; Frankel, Brown, & Burdon, 1995b) and overlapped with, or were located in the vicinities of, areas sustaining suitable habitat conditions for the species during different periods of time since the LGM*.* Cluster 2 may have originated in the northern Caribbean region as testified by high LCA richness values found in the buffer zone or Tayrona National Park (ZAT), and migrated southward and eastward toward the Chicamocha Canyon (CHI) during the period of range expansion of *A saman* predicted during the LGM. However, our data do not exclude the possibility of an opposite scenario either. Clusters 1 and 3 on the other hand may have formed through prolonged isolation in the middle and upper Cauca river valley around ITU and PAI‐MAT, were favorable climate conditions are likely to have persisted since the LGM or longer. The fact that most populations along the Cauca river valley, as well as those in the Caribbean region contained individuals of mixed origin may–at least partly—be due to human‐influenced movement of planting stock initiated after the European colonization.

Natural seed dispersal of *A. saman* is carried out by rodents, tapirs, and peccaries (Allen & Allen, 1981; Durr, 2001). However, in prehistoric times now extinct Pleistocene horses may also have been important, a role which today is likely to have been taken over by domesticated horses and cows (Janzen & Martin, 1982). Due to *A. saman's* popularity as a shade tree in pastures and farm land since the initiation of the European colonization and possibly before, reproductive material has been distributed extensively through human intervention, either as seeds or seedlings, or as seeds in the gut of domestic animals. As cattle breeding in Colombia became significant only in the seventeenth century (Etter, 2015), it is unlikely that the cultivation or human movement of *A.saman* for providing shade (and fodder) to cattle would have started much earlier. The largest *A. saman* trees we measured were found at La Paila (PAI) (Figure S2). All trees with DBH 3.5–4.5 m (which might be more than 400 years old; CABI,^2018^) were assigned to cluster 3, suggesting that this cluster might have originated in the upper Cauca river valley and hence cluster 1 in the middle Cauca river valley (Figure 5). Thomas et al. (2017b) similarly identified the Paila area as the putative origin of a genetic cluster of *Enterolobium cyclocarpum*, a tree species with analogous growth form and human use as *A. saman*.

Interestingly, the trees we sampled in Tatacoa (TAT) in the upper Magdalena river valley pertained to genetic groups with putative origin in the Cauca river valley and the Caribbean region. This might indicate that these trees were established from germplasm introduced by human activities. However, the possibility that some of the genetic groups arrived there through natural range expansion processes in prehistoric times cannot be ruled out. Our LGM suitability model suggests that the upper Magdalena river valley may have been connected by a continuous suitable area to the Chicamocha canyon and the Caribbean region, facilitating the arrival of cluster 2. On the other hand, a dispersal route across the Andes, connecting the Cauca and Magdalena river valleys might also have existed at one point in prehistory. Cross‐Andean dispersal has been documented previously for SDTF species (Quintana, Pennington, Ulloa, & Balslev, 2017), and the Colombian interAndean valleys are floristically more similar than other SDTF regions (González‐M et al., 2018). Bocanegra‐González et al. (2018) reported on a similar genetic pattern for *Ceiba pentandra* as we found for *A. saman* alluding to the possibility of cross‐Andean dispersal, especially because it is less likely that the trees they sampled there established through human intervention. However, as mentioned above, these interpretations have to be treated with caution, and more exhaustive sampling is needed to confirm or refute their validity.

Cluster 4 is likely to have originated in the Patia river valley (PAT; Figure 5) where *A. saman* populations seem to have been completely isolated from populations elsewhere in the country since the LGM or longer, as supported by the high pairwise F_ST_ values (Table S6). This group also showed the lowest levels of diversity of all sampling areas, in line with similar findings for *Ceiba pentandra* (Bocanegra‐González et al., 2018). This is likely the consequence of long‐lasting processes of genetic isolation exacerbated by more recent impact of anthropogenic degradation.

The absence of signs of inbreeding in nearly all localities we sampled, despite centuries of vegetation degradation, might be due to the combination of the admixed nature of populations (Figure 5) and the effective gene flow occurring between trees even in a fragmented landscape mosaic. *Albizia saman* is self‐incompatible and pollinated by moths in the Sphingidae family which are able of crossing distances >500 m and visiting several trees during one night (Cascante et al., 2002; Haber & Frankie, 1989). Only the trees sampled at the SENA experimental station yielded a significantly positive inbreeding coefficient, which suggests that most of these trees may have been established from inbred seeds. Remarkably, the least diverse of our sampling sites, the Patia river valley (PAT), showed an excess of heterozygosity, suggesting high levels of cross‐pollination, at least at the time of establishment of these trees.

However, our assessments of inbreeding coefficient are based on samples from trees of different ages (likely tens to hundreds of years old), which might obscure potential negative effects of more recent fragmentation on progeny. Cascante et al. (2002) investigated the effect of habitat fragmentation of Costa Rican SDTF on the reproductive success, progeny vigor, and genetic variation of *A. saman*. While they did not detect any apparent decline in expected heterozygosity, allelic frequency and fruit yield in isolated *A. saman* trees, the progeny of isolated trees did show decreases in survival and vigor, as well as increases in inbreeding coefficient and self‐fertilization compared with trees form continuous populations. Future research should therefore assess potential effects of fragmentation on *A. saman* progeny in Colombian SDTF.

Despite the potential negative consequences of fragmentation, Cascante et al. (2002) also found that levels of cross‐pollination, multiple paternity, and reproductive capacity within isolated trees remained high enough to support their use and maintenance in human‐dominated landscapes. The employment of *A. saman* in silvopastoral and other agroforestry systems is bound to increase considering the huge pledges made by countries worldwide to restore degraded lands such as Initiative 20 × 20. This initiative aims to initiate the restoration of 20 million hectares of degraded lands in Latin America and the Caribbean by 2020, among others through the use of agroforestry (http://www.wri.org/our-work/project/initiative-20920). Our finding that most of currently suitable areas for *A. saman* are expected to remain so under climate change, or might even expand, supports its use in future tree planting efforts.

To promote the good performance and long‐term persistence of planted *A. saman* tree stand, it will be critical to ensure that planting material has a sufficiently broad genetic basis and is adapted to the current and expected future environmental conditions at each planting site (Jalonen, Valette, Boshier, Duminil, & Thomas, 2018; Roshetko et al., 2017). For ensuring genetic diversity in planting stock, source populations should be large (ideally 100–500 reproductively mature individuals), and seeds should be obtained from a high number (>15) of mother trees per population (Bozzano et al., 2014). The selection of germplasm that is adapted to different planting conditions is not always trivial and requires knowledge on the nature of genotype‐by‐environment (GxE) interactions, which is typically derived from provenance and progeny trials (Thomas et al., 2014). Such trails for *A. saman* currently do not exist or are not mature enough in Colombia to guide decision making. In the absence of GxE data, the genetic groups we identified here can serve as a first entry point to guide the selection of adapted planting materials and avoid genetic pollution (Azpilicueta et al., 2013; Thomas et al., 2017a). Particularly in areas where all or most trees we sampled pertained to one single genetic cluster, such as the Patia (PAT) and Chicamocha (CHI) river valleys as well as Tayrona National Park of which we only sampled the buffer zone (ZAT), germplasm should be sourced from a high number of local trees. In the geographical range of the populations containing genotypes of mixed origin (the Cauca and upper Magdalena river valleys and the Caribbean region excluding Tayrona National Park and its bufferzone), germplasm can be used from any of the prevailing genetic clusters. As a word of caution, reproductive material of the tree collection in the experimental station SENA, which scored high for the inbreeding coefficient, should be mixed with materials from nearby sources within the Caribbean region such as Zambrano.

Viable populations of *A. saman* have to be identified as sources of germplasm in each of the regions where the four clusters are likely to have originated, and be the focus of in situ conservation measures. Such populations should best have high genetic diversity, show absence of inbreeding, and be located in areas where habitat conditions are expected to remain suitable in the future. Recommended areas for this would be the Chicamocha river valley (CHI) combined with the Tayrona National Park (ZAT) for cluster 2, la Paila (PAI) for cluster 3, Santa Fé‐Cotove (SFE‐COT) for cluster 2, and the Patia river valley (PAT) for cluster 4. Although populations in the Chicamocha river valley and the Tayrona National Park pertain to the same genetic group, they are geographically isolated and genetically differentiated enough (*F*_ST_ = 0.09; Table S6), to justify parallel conservation efforts. While the genetic diversity of trees sampled in Patía (PAT) was substantially lower than that of all other sites, their genetic uniqueness justifies the implementation of long‐term conservation strategies. Furthermore, our findings for *A. saman* mirror the previously reported genetic uniqueness of *C. pentandra* trees in the Patia river valley which might point to a broader biogeographical pattern, calling for the urgent establishment of the Patía Fauna and Flora Sanctuary conservation unit proposed by the Colombian national system of national parks (PNNC, 2018).

## CONFLICT OF INTEREST

None declared.

## AUTHOR CONTRIBUTIONS

ET, LGMH, and CAC designed the study; CAAM, CAC, and LGMH carried out field work; CAAM and JG carried out laboratory work; ET and CAAM performed data curation and statistical analyses and prepared the first draft of the manuscript. All authors contributed to revisions of the manuscript.

## Data Availability

Genetic characterization data are available via the following Dryad link: https://datadryad.org/stash/share/Wf0fYPyCWS-S03-ERR53f3BXljXd3nxASmytFSnVGIA; https://doi.org/10.5061/dryad.kh1893223

## References

[ece36005-bib-0001] Aguilar, R. , Ashworth, L. , Galetto, L. , & Aizen, M. A. (2006). Plant reproductive susceptibility to habitat fragmentation: Review and synthesis through a meta‐analysis. Ecology Letters, 9(8), 968–980. 10.1111/j.1461-0248.2006.00927.x 16913941

[ece36005-bib-0002] Aguilar, R. , Quesada, M. , Ashworth, L. , Herrerias‐diego, Y. , & Lobo, J. (2008). Genetic consequences of habitat fragmentation in plant populations: Susceptible signals in plant traits and methodological approaches. Molecular Ecology, 17(24), 5177–5188. 10.1111/j.1365-294X.2008.03971.x 19120995

[ece36005-bib-0003] Allen, O. N. , & Allen, E. K. (1981). The Leguminosae. A source book of characteristics, uses and nodulation. Madison, USA: University of Wisconsin Press.

[ece36005-bib-0004] Alzate‐Marin, A. L. , Guidugli, M. C. , Soriani, H. H. , Martinez, C. A. , & Mestriner, M. A. (2009). An efficient and rapid DNA minipreparation procedure suitable for PCR/SSR and RAPD analyses in tropical forest tree species. Brazilian Archives of Biology and Technology, 52(5), 1217–1224. 10.1590/S1516-89132009000500020

[ece36005-bib-0005] Azpilicueta, M. M. , Gallo, L. A. , van Zonneveld, M. , Thomas, E. , Moreno, C. , & Marchelli, P. (2013). Management of Nothofagus genetic resources: Definition of genetic zones based on a combination of nuclear and chloroplast marker data. Forest Ecology and Management, 302, 414–424. 10.1016/j.foreco.2013.03.037

[ece36005-bib-0006] Banda‐R, K. , Delgado‐Salinas, A. , Dexter, K. G. , Linares‐Palomino, R. , Oliveira‐Filho, A. , Prado, D. , … Pennington, R. T. (2016). Plant diversity patterns in neotropical dry forests and their conservation implications. Science, 353(6306), 1383–1387. 10.1126/science.aaf5080 27708031

[ece36005-bib-0007] Bocanegra‐González, K. T. , Thomas, E. , Guillemin, M. L. , Alcázar Caicedo, C. , Moscoso Higuita, L. G. , Gonzalez, M. A. , & Carvalho, D. D. (2019). Diversity and genetic structure of four keystone trees species of the Colombian tropical dry forest. Caldasia, 41, 78–91.

[ece36005-bib-0008] Bocanegra‐González, K. T. , Thomas, E. , Guillemin, M.‐L. , de Carvalho, D. , Gutiérrez, J. P. , Alcázar Caicedo, C. , … González, M. A. (2018). Genetic diversity of *Ceiba pentandra* in Colombian seasonally dry tropical forest: Implications for conservation and management. Biological Conservation, 227, 29–37. 10.1016/j.biocon.2018.08.021

[ece36005-bib-0009] Bozzano, M. , Jalonen, R. , Thomas, E. , Boshier, D. , Gallo, L. , Cavers, S. , … Loo, J. (2014). Genetic considerations in ecosystem restoration using native tree species. State of the World’s Forest Genetic Resources – Thematic Study. Rome, Italy: FAO and Bioversity International Retrieved from http://www.fao.org/publications/card/en/c/4f411455-6411-4319-8336-e49fab43c416/

[ece36005-bib-0010] CABI (2013). The CABI Encyclopedia of Forest Trees. Wallingford, UK: CABI Publishing.

[ece36005-bib-0011] CABI (2018). Samanea saman In Invasive species compendium. Wallingford, UK: CAB International Retrieved from http://www.cabi.org/isc

[ece36005-bib-0012] Caetano, S. , Prado, D. , Pennington, R. T. , Beck, S. , Oliveira‐Filho, A. , Spichiger, R. , & Naciri, Y. (2008). The history of Seasonally Dry Tropical Forests in eastern South America: Inferences from the genetic structure of the tree *Astronium urundeuva* (Anacardiaceae). Molecular Ecology, 17(13), 3147–3159. 10.1111/j.1365-294X.2008.03817.x 18522691

[ece36005-bib-0013] Cascante, A. , Quesada, M. , Lobo, J. J. , & Fuchs, E. A. (2002). Effects of dry tropical forest fragmentation on the reproductive success and genetic structure of the tree *Samanea saman* . Conservation Biology, 16(1), 137–147. 10.1046/j.1523-1739.2002.00317.x 35701973

[ece36005-bib-0014] Collevatti, R. G. , Terribile, L. C. , Lima‐Ribeiro, M. S. , Nabout, J. C. , de Oliveira, G. , Rangel, T. F. , … Diniz‐Filho, J. A. F. (2012). A coupled phylogeographical and species distribution modelling approach recovers the demographical history of a Neotropical seasonally dry forest tree species. Molecular Ecology, 21(23), 5845–5863. 10.1111/mec.12071 23094833

[ece36005-bib-0015] Dent, E. A. , & VonHoldt, B. M. (2011). STRUCTURE HARVESTER: A website and program for visualizing STRUCTURE output and implementing the Evanno method. Conservation Genetics Resources, 4(2), 359–361. 10.1007/s12686-011-9548-7

[ece36005-bib-0016] Doyle, J. J. , & Doyle, J. L. (1990). Isolation of plant DNA from fresh tissue. Focus, 12, 13–15.

[ece36005-bib-0017] Duminil, J. , Fineschi, S. , Hampe, A. , Jordano, P. , Salvini, D. , Vendramin, G. G. , & Petit, R. J. (2007). Can population genetic structure be predicted from life‐history traits? The American Naturalist, 169(5), 662–672. 10.1086/513490 17427136

[ece36005-bib-0018] Durr, P. A. (2001). The biology, ecology and agroforestry potential of the raintree, *Samanea saman* (Jacq.) Merr. Agroforestry Systems, 51(3), 223–237. 10.1023/A:1010765022497

[ece36005-bib-0019] Escalante, E. (1997). Saman (*Albizia saman*) in agroforestry systems in Venezuela In Zabala N. (Ed.), Proceedings of International Workshop on Albizia and Paraserianthes Species (pp. 93–97). Bislig, Phillipines.

[ece36005-bib-0020] Etter, A. (2015) La transformaciones del uso de la tierra y los ecosistemas durante el período colonial en Colombia In Meisel Roca A., & Ramírez G. M. T. (Eds.), La economía colonial de la Nueva Granada (primera ed., pp. 62–103). Bogota D.C. Colombia: FCE, Banco de la República 10.1017/CBO9781107415324.004

[ece36005-bib-0021] Etter, A. , McAlpine, C. , & Possingham, H. (2008). Historical patterns and drivers of landscape change in Colombia Since 1500: A regionalized spatial approach. Annals of the Association of American Geographers, 98(1), 2–23. 10.1080/00045600701733911

[ece36005-bib-0022] Evanno, G. , Regnaut, S. , & Goudet, J. (2005). Detecting the number of clusters of individuals using the software STRUCTURE: A simulation study. Molecular Ecology, 14(8), 2611–2620. 10.1111/j.1365-294X.2005.02553.x 15969739

[ece36005-bib-0023] Excoffier, L. , Smouse, P. E. , & Quattro, J. M. . (1992). Analysis of molecular variance inferred from metric distances among DNA haplotypes: Application to human mitochondrial DNA restriction data. Genetics, 131(2), 479–491. 1644282 10.1093/genetics/131.2.479 PMC1205020

[ece36005-bib-0024] Frankel, O. H. , Brown, A. H. D. , & Burdon, J. (1995a). The conservation of cultivated plants In The conservation of plant biodiversity (1st ed., pp. 79–117). Cambridge, UK: Cambridge University Press.

[ece36005-bib-0025] Frankel, O. H. , Brown, A. H. D. , & Burdon, J. (1995b). The genetic diversity of wild plants In The conservation of plant biodiversity (1st ed., pp. 10–38). Cambridge, UK: University Press.

[ece36005-bib-0026] García, H. , Corzo, G. , Isaacs, P. , & Etter, A. (2014). Distribución y estado actual de los remanentes del Bioma de Bosque Seco Tropical en Colombia: insumos para su gestión In Pizano C., & García H. (Eds.), El Bosque seco tropical en Colombia (1st ed.), pp. 228–251).. Bogota D.C. Colombia: Instituto de Investigación de Recursos Biológicos Alexander von Humboldt.

[ece36005-bib-0027] Gonzalez, M. , & Quintero, L. (2017). 'Plantas' In Gonzalez M., & Arenas-Castro H. (Eds.), Recolección de tejidos biológicos para análisis genéticos (p. 11). Bogota D.C. Colombia: Instituto de Investigación de Recursos Biológicos Alexander von Humboldt.

[ece36005-bib-0028] González‐M, R. , García, H. , Isaacs, P. , Cuadros, H. , López‐Camacho, R. , Rodríguez, N. , … Pizano, C. (2018). Disentangling the environmental heterogeneity, floristic distinctiveness and current threats of tropical dry forests in Colombia. Environmental Research Letters, 13, 045007 10.1088/1748-9326/aaad74

[ece36005-bib-0029] Haber, W. A. , & Frankie, G. W. (1989). A tropical hawkmoth community: Costa Rican dry forest Sphingidae. Biotropica. JSTOR, 155–172. 10.2307/2388706

[ece36005-bib-0030] Hengl, T. , de Jesus, J. M. , MacMillan, R. A. , Batjes, N. H. , Heuvelink, G. B. M. , Ribeiro, E. , … Gonzalez, M. R. (2014). SoilGrids1km — Global soil information based on automated mapping. PLoS ONE, 9(8), e105992 10.1371/journal.pone.0105992 25171179 PMC4149475

[ece36005-bib-0031] Hijmans, R. J. (2012) Cross‐validation of species distribution models: removing spatial sorting bias and calibration with a null model. Ecology, 93(3), 679–688. 10.1890/11-0826.1 22624221

[ece36005-bib-0032] Hijmans, R. J. , Cameron, S. E. , Parra, J. L. , Jones, P. G. , & Jarvis, A. (2005). Very high resolution interpolated climate surfaces for global land areas. International Journal of Climatology, 25, 1965–1978. 10.1002/joc.1276

[ece36005-bib-0033] Jalonen, R. , Valette, M. , Boshier, D. , Duminil, J. , & Thomas, E. (2018). Forest and landscape restoration severely constrained by a lack of attention to the quantity and quality of tree seed: Insights from a global survey. Conservation Letters, 11(4), e12424 10.1111/conl.12424

[ece36005-bib-0034] Janzen, D. H. (1988). Tropical dry forests, The most endangered major tropical ecosystem In Wilson E. O., & Peter F. M. (Eds.), Biodiversity (pp. 130–137). Washington, DC: National Academy of Sciences/Smithsonian Institution.

[ece36005-bib-0035] Janzen, D. H. , & Martin, P. S. (1982). Neotropical anachronisms: The fruits the gomphotheres ate. Science, 215(4528), 19–27. 17790450 10.1126/science.215.4528.19

[ece36005-bib-0036] Jombart, T. (2008). adegenet: A R package for the multivariate analysis of genetic markers. Bioinformatics (Oxford, England), 24(11), 1403–1405. 10.1093/bioinformatics/btn129 18397895

[ece36005-bib-0037] Kamvar, Z. N. , Tabima, J. F. , & Grünwald, N. J. (2014). Poppr: An R package for genetic analysis of populations with clonal, partially clonal, and/or sexual reproduction. PeerJ. 2, e281 10.7717/peerj.281 24688859 PMC3961149

[ece36005-bib-0038] Kasthurirengan, S. , Xie, L. , Li, C. H. , Fong, Y. K. , & Hong, Y. (2013). In vitro propagation and assessment of genetic stability of micropropagated Samanea saman (rain tree) using microsatellite markers. Acta Physiologiae Plantarum, 35(8), 2467–2474. 10.1007/s11738-013-1281-2

[ece36005-bib-0039] Kindt, R. (2018). Ensemble species distribution modelling with transformed suitability values. Environmental Modelling & Software, 100, 136–145. 10.1016/j.envsoft.2017.11.009

[ece36005-bib-0040] Leonard, B. E. , & Sherratt, H. S. A. (1967). The investigation of tropical medicinal plants: Pharmacological properties of some alkaloids from *Pithecolobium saman* and *Strychnos toxifera* . Tropical Science, 9, 122–135.

[ece36005-bib-0041] Lowe, A. J. , Boshier, D. , Ward, M. , Bacles, C. F. E. , & Navarro, C. (2005). Genetic resource impacts of habitat loss and degradation; reconciling empirical evidence and predicted theory for neotropical trees. Heredity, 95(4), 255–273. 10.1038/sj.hdy.6800725 16094300

[ece36005-bib-0042] Lowe, A. J. , Breed, M. F. , Caron, H. , Colpaert, N. , Dick, C. , Finegan, B. , … Cavers, S. (2018). Standardized genetic diversity‐life history correlates for improved genetic resource management of Neotropical trees. Diversity and Distributions, 24, 730–741. 10.1111/ddi.12716

[ece36005-bib-0043] Marchelli, P. , Thomas, E. , Azpilicueta, M. M. , van Zonneveld, M. , & Gallo, L. (2017). Integrating genetics and suitability modelling to bolster climate change adaptation planning in Patagonian Nothofagus forests. Tree Genetics & Genomes, 13, 119.

[ece36005-bib-0044] Mayle, F. E. , Beerling, D. J. , Gosling, W. D. , & Bush, M. B. (2004). Responses of Amazonian ecosystems to climatic and atmospheric carbon dioxide changes since the last glacial maximum. Philosophical Transactions of the Royal Society of London. Series B, Biological Sciences, 359(1443), 499–514. 10.1098/rstb.2003.1434 15212099 PMC1693334

[ece36005-bib-0045] Miles, L. , Newton, A. C. , DeFries, R. S. , Ravilious, C. , May, I. , Blyth, S. , … Gordon, J. E. (2006). A global overview of the conservation status of tropical dry forests. Journal of Biogeography, 33(3), 491–505. 10.1111/j.1365-2699.2005.01424.x

[ece36005-bib-0046] Murphy, P. G. , & Lugo, A. E. (1986). Ecology of tropical dry forest. Annual Review of Ecology and Systematics, 17(1), 67–88. 10.1146/annurev.es.17.110186.000435

[ece36005-bib-0047] Nei, M. (1973). Analysis of gene diversity in subdivided populations. Proceedings of the National Academy of Sciences of the United States of America, 70(12), 3321–3323. 10.1073/pnas.70.12.3321 4519626 PMC427228

[ece36005-bib-0048] Novaes, R. M. , Rodrigues, J. G. , & Lovato, M. B. (2009). An efficient protocol for tissue sampling and DNA isolation from the stem bark of Leguminosae trees. Genetics and Molecular Research, 8, 86–96. 10.4238/vol8-1gmr542 19283676

[ece36005-bib-0049] Pennington, T. R. , Prado, D. E. , & Pendry, C. A. (2000). Neotropical seasonally dry forests and Quaternary vegetation changes. Journal of Biogeography, 27(2), 261–273. 10.1046/j.1365-2699.2000.00397.x

[ece36005-bib-0050] Pizano, C. , Cabrera, M. , & García, H. (2014). El Bosque Seco Tropical en Colombia. Generalidades y Contexto In Pizano C., & García H. (Eds.), Bosque seco tropical en Colombia (1st ed., pp. 36–47). Bogotá, D.C. Colombia: Instituto de Investigación de Recursos Biológicos Alexander von Humboldt.

[ece36005-bib-0051] PNNC (Parques Nacionales Naturales de Colombia) (2018) Portafolio de nuevas áreas protegidas. Retrieved from http://www.parquesnacionales.gov.co/portal/es/sistema-nacional-de-areas-protegidas-sinap/portafolio-de-nuevas-areas-protegidas-del-sistemas-de-parques-nacionales/ (last consulted 15‐9‐2018).

[ece36005-bib-0052] Prado, D. , & Gibbs, P. (1993). Patterns of species distributions in the dry seasonal forests of South America. Annals of the Missouri Botanical Garden, 80(4), 902–927. 10.1086/331357

[ece36005-bib-0053] Pritchard, J. K. , Stephens, M. , & Donnelly, P. (2000). Inference of population structure using multilocus genotype data. Genetics, 155, 945–959. 10.1111/j.1471-8286.2007.01758.x 10835412 PMC1461096

[ece36005-bib-0054] Quintana, C. , Pennington, R. T. , Ulloa, C. U. , & Balslev, H. (2017). Biogeographic barriers in the andes: Is the Amotape—Huancabamba zone a dispersal barrier for dry forest plants? Annals of the Missouri Botanical Garden, 102(3), 542–550. 10.3417/D-17-00003A

[ece36005-bib-0055] Ramirez Villegas, J. , & Jarvis, A. (2010). Downscaling global circulation model outputs: The delta method. Cali, Colombia: International Center for Tropical Agriculture (CIAT).

[ece36005-bib-0056] Roshetko, J. (1995). Albizia saman: pasture improvement, shade, timber and more In NFTA 95‐02. Nitrogen Fixing Tree Association (p. 2). Morrilton, AR: Winrock International.

[ece36005-bib-0057] Roshetko, J. M. , Dawson, I. K. , Urquiola, J. , Lasco, R. D. , Leimona, B. , Weber, J. C. , … Jamnadass, R. (2017). To what extent are genetic resources considered in environmental service provision? A case study based on trees and carbon sequestration. Climate and Development, 10(8), 755–768. 10.1080/17565529.2017.1334620

[ece36005-bib-0058] Schuelke, M. (2000) An economic method for the fluorescent labeling of PCR fragments. Nature Biotechnology, 18(2), 233–234. 10.1038/72708. 10657137

[ece36005-bib-0059] Subansenee, W. (1994) Economic value of Albizia saman In Raintree J., & Francisco H. (Eds.), Marketing of Multipurpose Tree Products in Asia: Proceedings of an International Workshop (pp. 229–235).

[ece36005-bib-0060] Thomas, E. , Alcazar, C. , Moscoso Higuita, L. G. , Osorio, L. F. , Salgado-Negret, B. , Gonzalez, M. , … Ramirez, W. (2017a). The importance of species selection and seed sourcing in forest restoration for enhancing adaptive potential to climate change: Colombian tropical dry forest as a model. CBD Technical Series, 89, 122–132.

[ece36005-bib-0061] Thomas, E. , Gil Tobón, C. , Gutiérrez, J. P. , Alcázar Caicedo, C. , Moscoso Higuita, L. G. , Becerra, L. A. , … González, M. A. (2017b). Genetic diversity of Enterolobium cyclocarpum in Colombian seasonally dry tropical forest: Implications for conservation and restoration. Biodiversity and Conservation, 26, 825–842. 10.1007/s10531-016-1274-8

[ece36005-bib-0062] Thomas, E. , Jalonen, R. , Loo, J. , Boshier, D. , Gallo, L. , Cavers, S. , … Bozzano, M. (2014). ‘Genetic considerations in ecosystem restoration using native tree species. Forest Ecology and Management, 333, 66–75. 10.1016/j.foreco.2014.07.015

[ece36005-bib-0063] Thomas, E. , van Zonneveld, M. , Loo, J. , Hodgkin, T. , Galluzzi, G. , & van Etten, J. (2012). Present spatial diversity patterns of *Theobroma cacao* L. in the neotropics reflect genetic differentiation in pleistocene refugia followed by human‐influenced dispersal. PLoS ONE, 7(10), e47676 10.1371/journal.pone.0047676 23112832 PMC3480400

[ece36005-bib-0064] Verbylaite, R. , Beisys, P. , Rimas, V. , & Kuusiene, S. (2010). Comparison of ten DNA extraction protocols from wood of European aspen (*Populus tremula* L.). Baltic Forestry, 16(1), 35–42.

[ece36005-bib-0065] Vina, A. , & Cavelier, J. (1999). Deforestation rates (1938–1988) of tropical lowland forests on the Andean foothills of Colombia1. Biotropica, 31(1), 31–36. 10.1111/j.1744-7429.1999.tb00114.x

[ece36005-bib-0066] Vitorino, L. C. , Lima‐Ribeiro, M. S. , Terribile, L. C. , & Collevatti, R. G. (2016). Demographical history and palaeodistribution modelling show range shift towards Amazon Basin for a Neotropical tree species in the LGM. BMC Evolutionary Biology. BMC Evolutionary Biology, 16(1), 213 10.1186/s12862-016-0779-9 27737632 PMC5062830

