# Supplementary material for: Genetic diversity of the rain tree (Albizia saman) in Colombian seasonally dry tropical forest for informing conservation and restoration interventions
Source: Ecol Evol. 2020 Feb 5;10(4):1905–16. doi: 10.1002/ece3.6005 (PMC7042685; doi:10.1002/ece3.6005)
Supplement: Supplementary file 1 [file ECE3-10-1905-s001.docx]

| **a)**   | **b)**  **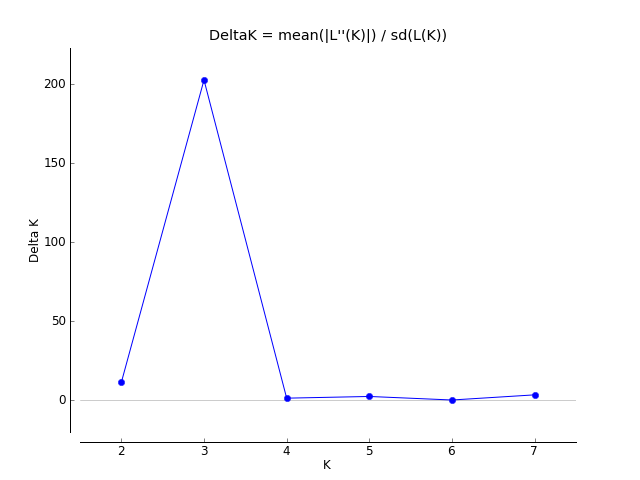** |
| --- | --- |

**c)**


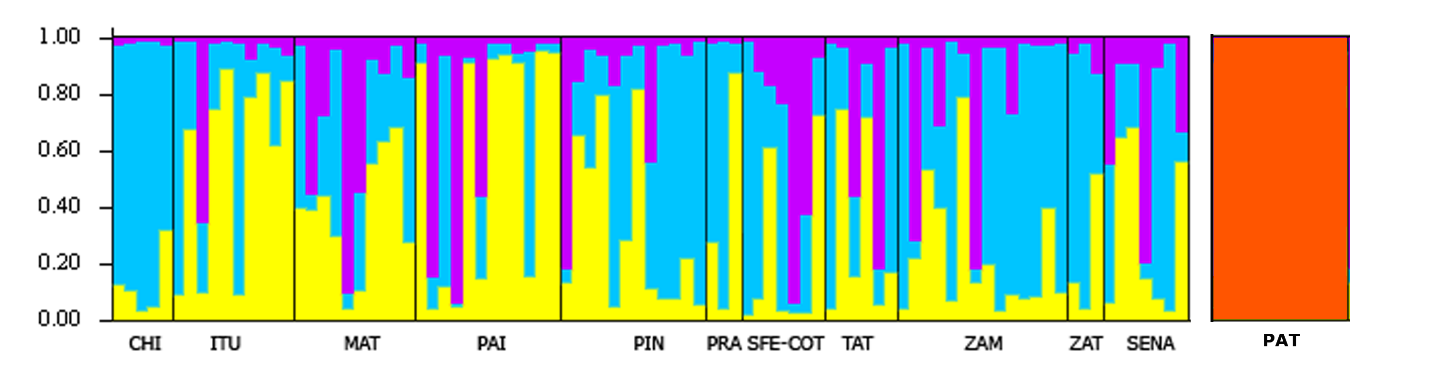


**Figure S1** Results of STRUCTURE analysis for *Albizia saman.* ΔK values computed for different values of K (1-8) for all samples (a) and all samples except those sampled at Patia (PAT; b); c**.** Bayesian admixture proportions of all sampled trees for K=4.
