# Supplementary material for: Genetic diversity of the rain tree (Albizia saman) in Colombian seasonally dry tropical forest for informing conservation and restoration interventions
Source: Ecol Evol. 2020 Feb 5;10(4):1905–16. doi: 10.1002/ece3.6005 (PMC7042685; doi:10.1002/ece3.6005)
Supplement: Supplementary file 2 [file ECE3-10-1905-s002.docx]

**Figure S2**. Diameter at breast height of *Albizia saman* trees plotted against the maximum membership to any of the clusters to which they are assigned. Please note that all trees with DBH >3.5m are from the Paila population and all have membership scores >0.75 for cluster 3 which might mean this cluster originated in this region.
