# Supplementary material for: Genetic diversity of the rain tree (Albizia saman) in Colombian seasonally dry tropical forest for informing conservation and restoration interventions
Source: Ecol Evol. 2020 Feb 5;10(4):1905–16. doi: 10.1002/ece3.6005 (PMC7042685; doi:10.1002/ece3.6005)
Supplement: Supplementary file 7 [file ECE3-10-1905-s007.docx]

**Table S5**. Suitability modelling metrics for *Albizia saman*

|  | | **Model calibration for projection to past** | **Model calibration for projection to future (2040-2069)** |
| --- | --- | --- | --- |
| **resolution** | | LGM: 2.5min (~4.625km)  Mid-Holocene: 30s (~925m) | 30s (~925m) |
| **Presence and background cells** | | 60 presence cells (151 sites); 7,282 background cells | 61 presence cells (151 sites), 8,070 background cells |
| **Explanatory variables** | | bios 2, 3, 4, 7, 8, 15, 18, 19 | Elevation, exposition, slope, terrain roughness index, direction of water flow, CEC, CLYPPT, CRFVOL, ORCDRC, SLTPPT, solar irradiation and bios 2, 3, 4, 7, 15, 18, 19 |
| **Best ensemble** | | Maxent, GBM, GLMstep, RPART, SVM | Maxent, GBM, RF, GAM, EARTH, RPART |
| **AUC** |  | 0.95 | 0.99 |
| **cAUC** |  | 0.71 | 0.76 |
| **maximum training sensitivity plus specificity threshold** | | 0.09 | 0.13 |

bio2= Mean Diurnal Range (Mean of monthly (max temp - min temp))
bio3 = Isothermality (bio2/bio7) (* 100)
bio4 = Temperature Seasonality (standard deviation *100)
bio7 = Temperature Annual Range (bio5-bio6)
bio8 = Mean Temperature of Wettest Quarter
bio15 = Precipitation Seasonality (Coefficient of Variation)
bio18 = Precipitation of Warmest Quarter
bio19 = Precipitation of Coldest Quarter
