# Supplementary material for: Genetic diversity of the rain tree (Albizia saman) in Colombian seasonally dry tropical forest for informing conservation and restoration interventions
Source: Ecol Evol. 2020 Feb 5;10(4):1905–16. doi: 10.1002/ece3.6005 (PMC7042685; doi:10.1002/ece3.6005)
Supplement: Supplementary file 8 [file ECE3-10-1905-s008.docx]

**Table S6** Pairwise Fst values (Nei 1973) for populations of *Albizia saman*. Values higher than 0.1 are highlighted in bold.

|  | CHI | SFE-COT | ITU | MAT | PAI | PIN | PRAD | TAT | ZAM | ZAT | SENA |
| --- | --- | --- | --- | --- | --- | --- | --- | --- | --- | --- | --- |
| SFE-COT | 0.044 | 0.000 |  |  |  |  |  |  |  |  |  |
| ITU | 0.050 | 0.050 | 0.000 |  |  |  |  |  |  |  |  |
| MAT | 0.041 | 0.037 | 0.034 | 0.000 |  |  |  |  |  |  |  |
| PAI | 0.052 | 0.041 | 0.042 | 0.030 | 0.000 |  |  |  |  |  |  |
| PIN | 0.037 | 0.035 | 0.037 | 0.026 | 0.036 | 0.000 |  |  |  |  |  |
| PRAD | 0.066 | 0.059 | 0.045 | 0.051 | 0.044 | 0.041 | 0.000 |  |  |  |  |
| TAT | 0.062 | 0.042 | 0.054 | 0.046 | 0.042 | 0.046 | 0.063 | 0.000 |  |  |  |
| ZAM | 0.033 | 0.028 | 0.039 | 0.032 | 0.038 | 0.035 | 0.033 | 0.032 | 0.000 |  |  |
| ZAT | 0.085 | 0.068 | 0.057 | 0.048 | 0.053 | 0.046 | **0.120** | 0.083 | 0.036 | 0.000 |  |
| SENA | 0.053 | 0.039 | 0.051 | 0.040 | 0.047 | 0.040 | 0.062 | 0.060 | 0.033 | 0.077 | 0.000 |
| PAT | **0.138** | **0.141** | **0.139** | **0.132** | **0.138** | **0.135** | **0.132** | **0.146** | **0.135** | **0.133** | **0.140** |
